# DNA replication stress: a source of APOBEC3B expression in breast cancer

**DOI:** 10.1186/s13059-016-1069-y

**Published:** 2016-09-30

**Authors:** David W. Cescon, Benjamin Haibe-Kains

**Affiliations:** 1 Princess Margaret Cancer Centre, University Health Network, Toronto, ON Canada; 2 Campbell Family Institute for Breast Cancer Research, Toronto, Canada; 3 Division of Medical Oncology and Hematology, Department of Medicine, University of Toronto, Toronto, Canada; 4 Department of Medical Biophysics, University of Toronto, Toronto, ON Canada; 5 Department of Computer Science, University of Toronto, Toronto, ON Canada; 6 Ontario Institute of Cancer Research, Toronto, ON Canada

## Abstract

APOBEC cytidine deaminases have been implicated as major contributors to the mutation burden in many cancers on the basis of their mutational signature. A new experimental study sheds light on the inciting factors, linking APOBEC3B expression to oncogene- and drug-induced replication stress.

**Electronic supplementary material:**

The online version of this article (doi:10.1186/s13059-016-1069-y) contains supplementary material, which is available to authorized users.

## Footprints in the genome: from patterns to processes

The Cancer Genome Atlas (TCGA) Project has been an undisputed success in advancing our knowledge of the many and varied subtypes of cancer. The identification of recurrent somatic alterations, patterns of gene expression and relationships between these have provided a crucial resource to inform our understanding of cancer biology and the development of biomarker-driven therapeutic strategies. Moreover, analysis of the types of mutations in these established cancers has revealed mutational signatures that reflect the processes that contributed to their genesis [1]. As expected, the mutational footprints of tobacco smoking and ultraviolet damage feature prominently in the cancers where epidemiologic links are established and well understood. More interesting, however, are those that were unanticipated, such as the pattern of base changes consistent with the mutagenic activity of the endogenous APOBEC3 cytidine deaminases. In a recently published study, Kanu and colleagues from the Swanton laboratory investigate the molecular basis of this phenomenon in breast cancer cell lines [2].

The APOBEC enzymes primarily function in innate antiviral immunity and deaminate cytosine to uracil in single-stranded DNA to generate C > T and C > G mutations in a preferential motif. Many solid tumors bear these genomic hallmarks and are believed to reflect the damage caused by one or more APOBECs. First identified in breast cancer datasets [3, 4], the mutagenic potential of the APOBEC family of enzymes had been recognized in preclinical systems, but the inferred mutational burden across a high proportion of tumor types represented a surprising discovery. Of the candidate APOBECs capable of such mutagenesis, APOBEC3B was fingered as the most likely culprit—in large part because of its overexpression observed in cancer. Following these discoveries, efforts quickly turned to the study of the upstream mechanisms of APOBEC dysregulation and the downstream consequences of this activity, in order to characterize this novel biology and pursue potential opportunities for cancer prevention or treatment.

The complexity of this undertaking is significant. The seven human APOBEC3 family members share significant homology, which limits the availability of reagents with high specificity. In addition, mice only have a single APOBEC3, meaning that mouse models have limited experimental application. Furthermore, the biology is complicated by the existence of a common human germline deletion polymorphism in APOBEC3B, which eliminates the entire coding region of this gene but is paradoxically associated with an *increased* risk of breast cancer [5] and an *increased* burden of APOBEC-pattern mutations [6, 7]. It has been suggested recently that APOBEC3A—rather than 3B—might be responsible for the bulk of APOBEC mutations in highly APOBEC-mutated tumors [7], but, in light of the low levels of APOBEC3A expression in epithelial cells, the mechanism for this remains unclear.

## Connecting replication stress and APOBEC3B through ATR

Kanu and colleagues focused on the association between replication stress and APOBEC3B activity in breast cancer cell lines, starting from the observation that the HER2-enriched subtype of breast cancers exhibits the greatest burden of APOBEC mutations [8] and the premise that oncogene-induced replication stress exposes single-stranded DNA (ssDNA) substrate susceptible to APOBEC mutagenesis [2]. They observe a trend of increased APOBEC3B, but not APOBEC3A or APOBEC3G, expression in HER2-enriched cell lines and demonstrate a correlation between the presence of markers of replication stress and both APOBEC3B expression and biochemical APOBEC3 deamination activity under basal conditions. Nucleoside supplementation to relieve replication stress resulted in a reduction in APOBEC3B expression and APOBEC3 activity, supporting this association. Treatment of the MCF10A non-tumorigenic breast epithelial cell line with nine DNA-damaging or anti-metabolite drugs followed by measurement of the ssDNA damage marker pS4/8 RPA identified a relationship between those that induced the highest levels of both APOBEC3B and RPA phosphorylation, although this was not entirely reproduced in the estrogen-receptor-positive MCF7 breast cancer cell line.

In their study, the authors also showed that the APOBEC3B upregulation following induction of replication stress is dependent on signaling through the ATR–CHK1 (checkpoint kinase 1) pathway, providing a mechanism for the observed phenomena. While not a primary focus of the paper, the authors examined the association between APOBEC3B expression and drug sensitivities. Interestingly, they noted a trend between APOBEC3B expression levels and in vitro sensitivity to the novel oral CHK1 inhibitor CCT244747 and speculated that markers of replication stress, including APOBEC3B, could be useful to predict response to this agent. While CCT244747 is not included in the recent GDSC1000 pharmacogenomic dataset, we did not observe a relationship between APOBEC3B expression and either AZD7762 or Calbiochem 681640, which inhibit CHK1 (Additional file 1: Supplementary methods; Additional file 2: Table S1), although the target selectivities of these three drugs do vary. By contrast, the authors observed no correlation between APOBEC3B expression and sensitivity to the DNA-damaging and antimetabolite drugs in their small panel of breast cancer cell lines—results we confirmed in GDSC1000 (Additional file 2: Table S1a, b). However, when considering all cancer types and cell lines, APOBEC3A and APOBEC3B expression was significantly associated with sensitivity to 16 and 38 drugs, respectively (false discovery rate <5 %, correlation adjusted for tissue type; Additional file 2: Table S1c, d), raising the possibility that expression of these genes could mediate or mark sensitivity to anti-cancer agents.

Together, this report presents an important contribution to the understanding of APOBEC3 dysregulation in cancer and is consistent with our previous observation that APOBEC3B is highly correlated with proliferation-related gene expression in large breast cancer datasets and across nearly all solid tumor types in TCGA [9]. The connection made here between replication stress and APOBEC upregulation highlights a particularly vulnerable context, where high levels of APOBEC3B enzyme and ssDNA substrate co-exist. It will be of great interest to see how generalizable the drug-induced upregulation of APOBEC3B is across cancer cell lines and to assess the impact of a broader group of drugs on APOBEC expression.

## Minding the As and Bs

There remain many unanswered questions that must be addressed in order to decipher the APOBEC puzzle and translate these discoveries towards the clinic. Importantly, evidence that upregulation of APOBEC3B contributes to therapeutic resistance or that its modulation improves disease control or anti-tumour activity of other agents is lacking. The widely reported associations between increased APOBEC3B expression and outcomes in patients are likely confounded by the fact that its expression reflects a proliferative state. Indeed, while expression levels point towards APOBEC3B as the guilty party, the increase in cancer risk among germline APOBEC3B deletion carriers [5] and the increased burden of APOBEC-pattern mutations [6] attributed to APOBEC3A [7] seen in these patients highlights key unresolved issues.

As Kanu and colleagues show, APOBEC3A is barely detectable in breast cancer cell lines and was not increased by the stimuli that induced APOBEC3B. Whether or how APOBEC3A, with its potent enzymatic activity, could be upregulated transiently under some condition to strike in a “hit-and-run” fashion is unknown. However, our analysis of breast cancer gene expression data shows clearly that the correlates of APOBEC3B expression in tumors (proliferation) are distinct from all of the other members of the APOBEC3 family, which are very strongly associated with a STAT1/interferon signature [9]. This observation, together with the experimental data reported here, suggests that the inciting triggers for APOBEC3A and APOBEC3B expression are likely to differ.

In conclusion, Kanu and colleagues provide important insights into our understanding of the APOBEC phenomenon. Their careful experimentation complements the growing body of genomics-based analyses that seek to fully decrypt how and why these immune defenders turn against our genomes. Well-conducted experimental studies and continued interrogation of the genomes of nascent and late-stage cancers, both before and following treatment, will undoubtedly reveal much about this enemy within and should clarify the potential of translating this knowledge for the prevention or treatment of cancers where APOBEC operates.

## Additional files

Additional file 1: Supplementary Methods [10]. (DOCX 14 kb) Additional file 2: Supplementary Table 1. (XLSX 68 kb)
