# Supplementary material for: DNA replication stress: a source of APOBEC3B expression in breast cancer
Source: Genome Biol. 2016 Sep 30;17:202. doi: 10.1186/s13059-016-1069-y (PMC5045630; doi:10.1186/s13059-016-1069-y)
Supplement: Additional file 1: — Supplementary Methods [10]. (DOCX 14 kb) [file 13059_2016_1069_MOESM1_ESM.docx]

**Supplementary Methods**

*Association between APOBEC3A/B expression and drug sensitivity*

We used our *PharmacoGx* platform [[10]](https://paperpile.com/c/zoxkd7/Brlq) to collect normalized RNA-seq expression profiles for 935 cancer cell lines from the Cancer Genomics Hub, and pharmacological profiles for 251 drugs (GDSC1000). RNA-seq data were processed with the Tuxedo protocol using the Ensembl Genome Reference Consortium release GRCh37. We summarized the drug dose–response curves into a single sensitivity measure, the area under the curve (AUC) metric, that combines both potency and efficacy of drug responses.

The correlation between APOBEC expression and drug sensitivity was estimated using a linear-regression model adjusted by tissue type. Gene expression and drug sensitivity values were standardized before regression analysis. *P* values were corrected for multiple testing using the false discovery rate (FDR) approach.
